# The quality of life of 50-70 years old patients with orthopedic spinal stenosis surgery. A follow-up study (descriptive study) 

**DOI:** 10.22088/cjim.14.4.703

**Published:** 2023

**Authors:** Mehdi Teimouri, Sahar Sadat Lalehzar, Niloofar Habibpor, Ali Andalib

**Affiliations:** 1 Department of Orthopedic Surgery, School of Medicine, Isfahan University of Medical Sciences, Isfahan, Iran

**Keywords:** Lumbar, Spinal stenosis, Quality of life, Surgical procedure, Population.

## Abstract

**Background:**

Background: Nowadays, surgical procedures are assessed based on the state of an individual. This study aimed to investigate the effect of lumbar spinal stenosis surgery on the patient’s quality of life and motor functions in Kashani and Alzahra Hospital in Esfahan.

**Methods:**

In the present cross-sectional study, 40 patients aged between 50-70 were respectively evaluated who underwent lumbar spine stenosis surgery in Al Zahra and Kashani Hospitals in Esfahan University of Medical Sciences, Esfahan, Iran, during 2020-2021. The SF-36 questionnaire was used as a research tool. The visual analog scale (VAS), and spine functional index (SFI), were measured initially before surgery and 6 months and 9 months after surgery.

**Results:**

The mean scores of the SF-36, SFI, and VAS scores questionnaire were 87.95±4.94, 21.38±1.24, 6.07±0.69 (p<0.001) before surgery, 89.77±5.25, 19.73±1.40, 5.37±1.56 (p<0.001) six months after surgery, and 94.70±5.34, 18.63±1.56, 4.57±0.81 (p<0.001) nine months after surgery, and all were significant. Improvement in the domains of general health, role-physical, role disorder due to impaired physical health, social function, emotional role, and bodily pain was evident. Also, the overall quality of life was enhanced but energy levels and role disorder due to impaired mental health showed no improvement.

**Conclusion:**

Not only does lumbar spinal stenosis surgery significantly improve the general health, role-physical, and the social function of the patients but also enhances their quality of life.

Lumbar spinal stenosis, widely known as LSS, commonly affects middle-aged individuals due to the narrowing of the spinal canal, which is encasing the nerve-endings, exerting pressure on the blood vessels and nerves in the canal. This condition is usually associated with age-related degeneration or occurs as a result of changes in joints like lumbar vertebrae, intervertebral joints, and intervertebral discs (1) The most common manifestation of LSS is neurogenic lameness (or pseudo-lameness) which is defined as intermittent pain that spreads to the buttocks, thighs, legs, and feet and causes weakness during standing or walking.

 The pain normally resolves by sitting, lying down, or arching the back whilst significantly restraining physical activities (2). Moreover, LSS contributes to the majority of the low back pain causes about 50% of the cases (3). The prevalence of LSS surgery with increasing age owing to the degenerative pathogenesis of the disease is rare in people under 50 and widespread in people over 60, which means affecting more than 70% of them (1, 3). 

In addition, the prevalence of LSS was estimated at 7.5 to 10 % in Japan and around 5.22% in the United States (3). According to a meta-analysis on LSS, the estimated prevalence of LSS based on clinical diagnosis criteria varied from 11% among the general population to 25 to 39% in the clinical population. Furthermore, radiological findings suggestive of LSS were discerned in 11% of the asymptomatic population, 38% of the general population, and varied between 15 to 32 percent of the clinical population. Non-surgical treatments such as steroidal anti-inflammatory drugs, NSAIDs, analgesics, and physiotherapy could be beneficial and effective only during the initial stages of the disease but once the condition worsens inflicting disabilities, surgical interventions become imperative (4). LSS is one the most commonly diagnosed spinal disorders which undoubtedly, deters quality of life and more often necessitates surgical interventions in elderly patients (5). Pain and poor quality of life are two debilitating consequences of LSS in the elderly (4). Ozdemir et al. reported poor quality of life in LSS patients in their research conducted in Istanbul (6). During a follow-up on patients who underwent surgical interventions one year later, surgical treatments showed greater efficacy in comparison with conservative treatments. The variety of existing surgical treatment modes alongside various conservative treatments makes it tough for the physician to choose one definitive mode of intervention for LSS. More extensive research is required to derive one standard mode of assessment of results to compare surgical treatment with conservative treatment (2). In recent decades, research has been mainly focused on patients' quality of life (7). Quality of life means the patient exhibits emotional, social, and physical health (8). Comprehending and evaluating the quality of life is mandatory for improving patients' symptoms, care and rehabilitation. Addressing patient issues based on the quality of life may help improve and modify care suiting their needs or reveal that certain treatments are of little or no use to patients. Also, quality of life assessment can be used to identify a range of other problems that patients face. Meanwhile, the information can be passed on to future patients to help them predict and understand the consequences of the disease and make better treatment choices. In addition, treated patients with an extended life span, long after the treatment had ended, may suffer from long-term effects. Such long-term issues will not be taken into consideration if the quality of life was not assessed. Quality of life assessment is also essential to make clinical decisions as it predicts treatment success and therefore determines prognosis. The aforementioned statements emphasize the need for routine assessment of the quality of life via clinical studies (7). 

Further still, surgical outcomes nowadays are increasingly assessed via individual patient feedback while many modern interventions are performed primarily to enhance patients' quality of life. Hence, the main aim of LSS treatment is to control pain, improve function and physical activity, and thereby improve quality of life. Quality of life measurement plays a key role during post-intervention follow-up sessions (6). At present, post-op quality of life evaluations is crucial for analyzing surgical adverse effects. According to the study by Ozdemir et al. in Istanbul, both surgical and expectant management reduced pain and increased walking distance (6). Another study by Kobayashi Zashiomi et al. in Japan on LSS surgical treatment reported reduced back pain and diminished overall pain and numbness in the legs with an improved quality of life (9). 

So far, the literature lacks enough studies about this issue in Iran and the various results due to the differences in living conditions, customs, and different expectations levels of Iranian people, arising the need to investigate the effect of LSS surgery on public health, motor function and the overall quality of life in our country. Therefore, the current study was designed to evaluate the spinal stenosis surgery effectiveness on quality of life, pain and function of spine in three period of time before and after surgery. 

## Methods

**The Study design: **The present cross-sectional study was comprised of patients who underwent LSS surgery in Al-Zahra and Kashani Hospitals in Isfahan during the years 2020-2021. Our study met all ethical standards of Isfahan University of Medical Sciences under the ethical code of IR.MUI.MED.REC.1400. 341. throughout the research. Out of 82 patients, around 42 were patients who, either did not consent to participate or did not cooperate during follow-up were excluded providing a final sample size of 40 patients between the age ranges of 50-70 years. The sampling method was random 

**Sample volume formula** **:** (d=10)

$N=\frac{{\left(Z_{1-\frac{\alpha }{2}}+Z_{1-\beta }\right)}^{2}(\delta_{1}^{2}+\delta_{2}^{2})}{d^{2}}$

**Inclusion and exclusion criteria: **The inclusion criteria involved patients of the age range of 50-70 years who underwent surgery for lumbar spinal canal stenosis during September 2020-March 2021 and were still alive till September 2021 for follow-up. The candidates for LSS surgery at the mentioned hospitals who had no other physical or emotional co-existing conditions were evaluated before surgery and 6 and 9 months after surgery. Patients with known pre-existing mental and physical conditions which affected physical activities, those who acquired a new illness or died during the study period, those who did not consent to study participation or did not cooperate for follow-up were excluded from the study. 

**Data collection: **All patients underwent laminectomy and 12 among them underwent fusion and placement of pedicle screws in addition to laminectomy. 20 patients had their procedure performed in one level and 14 patients in 2 levels whilst 6 patients underwent the procedure in more than 3 levels.

The participants of our study were evaluated using a questionnaire in terms of pain, physical activity, mental health, and personal satisfaction. The tool used was a short form of general health status SF-36 with a score of zero to 121, while a full score of 121 indicated optimal health and quality of life in eight dimensions such as physical function, physical limitation, physical pain, general well-being, social functioning, mental health issues, and general mental health. This questionnaire has become the norm in our country. Validity and Reliability of the Short Form- 36 Items Questionnaire as a Measure of Quality of Life in Elderly Iranian Population was done in 2006 (10).

Also, we have followed our patients with spinal functional index questioner (SFI_score) and evaluated their pain with a VAS score. Validity and reliability of the VAS score and SFI score items questionnaire respectively as a measure of pain and function of spine in Iranian Population was done in 2007 and 2018.

The interviewer who was a medical student, presented at Kashani and Alzahra Subspecialized orthopedic clinic. The interview was conducted in person. She was assured full confidentiality of patient information, after debriefing the participants on the interview process and obtaining their informed consent. In case the patient was illiterate, the patient's companion would answer on behalf of the patient. Patients were divided into two groups, before surgery, 6 and 9 months after surgery. The face-to-face interview was performed by a medical student 6 and 9 months after the operation at the Kashani and Alzahra Hospitals’special orthopedic clinic. The scores of the questionnaire from each group were compared and descriptive data were extracted. SPSS software Version 23 and the paired-t test were utilized for data analysis. Values less than 0.05 were considered significant. 

**Statistical analysis: **The obtained data were analyzed using the Statistical Package for Social Sciences (SPSS) software (Version 24.0; SPSS Inc., Chicago, IL, USA). Demographic and clinical characteristics of patients were reported as frequency (percentage) for qualitative variables and mean± standard deviation (SD) for quantitative variables. Qualitative variables between the study groups were compared using the chi-squared test and Fisher's exact test. Normality of distribution in quantitative variables was assessed using the Shapiro Wilk test. Normally distributed quantitative variables were compared between the study groups using the independent t-test,paired t test and repeated measures Anova.

## Results

The present study performed on 40 patients consisted of 17 (42.5%) men and 23 (57.5%) women aged 50 to 70 years by the mean age of 63.05+5.02. All patients underwent laminectomy and 12 among them underwent fusion and placement of pedicle screws in addition to laminectomy. 20 patients had their procedure performed in one level and 14 patients in 2 levels whilst 6 patients underwent the procedure in more than 3 levels. The sf36 questionnaire examined eight domains, the results of which are depicted in the table (table 1). The mean total score before surgery was 87.95±4.94, 6-month post-surgery was 89.77±5.25, and 9-month after surgery was 94.70±5.34 and all had significant differences not only between groups in the simultaneous comparison of three groups but among all during the time (p<0.001). It means that changes of SF36 score over time are significant. Also, we compared each period of time two by two. In comparison between SF36 score before surgery with 6-months (P=0.001) and 9-months after that (p<0.001), 6-months after surgery with before (P=0.001) and 9-months after that, and 9-months after surgery before (p<0.001) and 6-months (p<0.001) after that, all were significant. It shows that in comparison of each period of times two by two, each period of time has significant relation with the time before and after it.

The SFI score was examined. The mean and standard deviation were 21.38±1.24 before surgery, 19.73±1.40 six months, and 18.63±1.56 nine-months after surgery. There was a significant relationship between all these times and also between groups (p<0.001) (table 2). Also, we compared each period of time two by two. In comparison between SFI score before surgery with 6-months (p<0.001) and 9-months after that (p<0.001), 6-months after surgery with before (p<0.001) and 9-months after that, and 9-months after surgery with before (p<0.001) and 6-months (p<0.001) after that, all were significant. It shows that in comparison of each period of times two by two, each period has significant relation with the time before and after it.

**Figure1:**
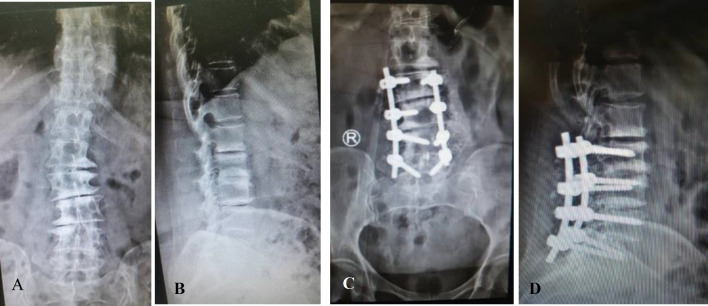
AP (A) and lateral (B) radiography of spine before surgery ,and AP (C)and lateral (D) radiography of spine after surgery

The VAS score was also examined to evaluate the pain before and after surgery. The mean and standard deviation were 6.07±0.69 before surgery, 5.37±1.56 six months, and, 4.57±0.81 nine months after surgery. There was a significant relationship between these times and also between groups (P=0.00) (table 3). Also, we compared each period of time two by two. In comparison between vas score before surgery with 6-months (p<0.001) and 9-months after that (p<0.001), 6-months after surgery with before (p<0.001) and 9-months after that, and 9-months after surgery with before (p<0.001) and 6 months (p<0.001) after that, all were significant. It shows that in comparison of each period of times two by two, each period has significant relation with the time before and after it. Pre- and postoperative radiographs were also obtained from these patients (figure 1). 

**Table 1:** SF36 QUESTIONER, all variable is presented as mean± SD, P-Values are reported according to the relevant tests which shows the compression of quality of life before, 6 and 9 months follow up after surgery

**P-value**	**9 months After surgery**	**6 months After surgery**	**Before surgery**	**Scope of study**
p<0.001 9.55±0.98 9.20±0.99 9.00±0.93				**general health** **(mean±SD)**
p<0.001	12.85±2.11	12.45±1.79	12.15±2.10	**Physical function** **(mean±SD)**
p<0.001	4.15±0.73	4.37±0.74	4.10±0.37	**Role disorder due to physical health** **(mean±SD)**
p<0.001	3.76±0.64	3.55±0.71	3.40±0.59	**Role disorder due to mental health** **(mean±SD)**
p<0.001	7.62±1.00	6.77±0.91	6.50±0.75	**Social function** **(mean±SD)**
P=0.051	33.57±4.17	33.22±3.68	32.55±3.38	**Emotional well-being** **(mean±SD)**
P=0.013	11.9±1.73	11.02±1.92	10.95±2.01	**Energy** **(mean±SD)**
p<0.001	10.02±0.94	9.17±0.54	9.40±0.49	**the pain** **(mean±SD)**
p<0.001	94.70±5.34	89.77±5.25	87.95±4.94	**Total**

**Table 2:** Spinal functional index (SFI). All variables are presented as mean± SD, P-Values are reported according to the relevant tests which is comparing the function of the spine before, 6 and 9 months after surgery

**P1**	**9month after surgery**	**6month after surgery**	**Before surgery**	
p<0.001	18.63±1.56	19.73±1.40	21.38±1.24	**SFI SCORE**

**Table 3:** The visual analog scale (VAS SCORE), all variable is presented as mean± SD, P-Values are reported according to the relevant tests which is comparing the pain before, 6 and 9 months after surgery

**P1**	**9month after surgery**	**6month after surgery**	**Before surgery**	
p<0.001	4.57±0.81	5.37±1.56	6.07±0.69	**VAS SCORE**

## Discussion

We compared spine surgery at 3 different times with VAS score, SF36 and SFI questionnaires. We found that spine surgery will significantly decrease the pain and increase the quality of life and function. Owing to the lifestyle, cultural diversity, and Iranian’s expectation level, we found that it is necessary to carry out the present study to determine the impact and efficacy of LSS surgery on the overall health and quality of life of the population and to examine if this mode of treatment will meet peoples' expectation. 

A considerable rise in the 36-SF score to 77.89 demonstrated that LSS surgery had positively impacted patients' quality of life. The positive effects were projected in the domains of general health, physical function, role disorders from impaired physical health, social function, emotional health, and pain. In concordance to our findings, the study by Eneqvist et al. conducted on 171 patients, who underwent LSS surgery, evaluated the quality of life of patients one year after surgery via EQ-5D and VAS EQ questionnaires depicted that surgery in conjunction with enhancing patients' quality of life, reduced pain, depression, and anxiety levels as well. These results were similar to our findings except for no effect on the patients' mental health. (11). In a study which measured the development and Validation of a Prediction Model for Pain and Functional Outcomes after lumbar spine surgery, the patients were mostly females comprising 57.5% of the study sample. The female to male ratio in terms of LSS prevalence in our study amounted to 1.35% similar to the study of Khor et al, who stated a high female to male ratio with 59.6% females (12). Likewise, studies led by Yüce İsmail et al. and Jansson et al. mentioned 53.6% and 53% female proportion in their studies respectively (13, 14). 

In another study in Washington, led by Khor et al, conducted on 1965 patients undergoing lumbar spinal surgery, about 1223 were diagnosed with LSS. These patients were evaluated one year after surgery via the PRO predictive tool which concluded the efficacy of the surgery in terms of reduced pain and improved physical activities (12). Furthermore, in the study by Yüce İsmail et al. on 918 patients undergoing LSS surgery, initially followed 6-months after surgery and then a year later, discerned an increase in the score 36-SF questionnaire and a decrease in the score of Oswestry Disability Index further confirming the improvement of symptoms and quality of life (11). Further confirming our results, the study by Hebert et al. on 548 patients during a post-surgical follow-up of 3, 12, and 24 months later using leg and back pain numeric rating scales and with aid of modified Oswestry disability index revealed a reduction in pain and patient disability after LSS surgery and around 29-42% of the patients either benefited less or not all from surgery (15). 

Besides the survey led by Jansson et al. consisting of 230 patients one year after LSS surgery using the EQ-5D questionnaire portrayed an improved quality of life with regards to health in 80% of the study participants. The study noted that only 27% of the study population were able to walk a distance of 500 meters at first, while the rate spiked up to 65% of the patients after surgery (13). 

In a clinical situation, one of the tools for measuring the quality of life after surgery is the VAS score. In one study, there were 383 patients from 5 unique studies. Meta-analysis of visual analog scale score for low back pain after surgery showed no significant difference at baseline (P = 0.49), at 2–3 months (P = 0.69), and the final follow-up (P = 0.26) (16, 17). Contrary to the results of the study in our study VAS score significantly decreased after surgery. In another report, a total of 21 eligible studies based on 2890 patients with degenerative LSS were included. The newer micro decompression technique (bilateral decompression via unilateral laminotomy (BDUL) performed better in decreasing the visual analog scale (VAS) score compared with conventional decompressive laminectomy (VAS score back pain, 1.22; 95% CI, 0.28–2.17; VAS score leg pain, 1.39; 95% CI, 0.82–1.96) (18).

Another example in agreement with the current study was the study led by Zarghooni et al. on 36 patients who underwent LSS surgery. The study used tools like EQ-5D, core outcome measures index (COMI), and Oswestry disability index 6 weeks, 12 months, and 12 months after surgery to evaluate the quality of life and the study found that the surgery caused a drastic reduction in pain and better quality of life even as early as 6 weeks. (16). It is noteworthy to emphasize that the present study observed significant improvements in domains like general health, physical function, pain, role disorders from impaired physical health, social function, and emotional health. On the whole, enhanced quality of life was observed while the energy levels of patients remained unchanged, which may be explained by their old age.

 In addition, role disorders from impaired mental health did not improve which may be due to differences in economy, living standards, conditions, social status, and given their old age. Our limitation in this study was low sample size and short duration of study. In conclusion, taking into consideration of the significant impact of LSS surgery on patients' quality of life while reducing patients' pain and physical disability, it is advisable to recommend this mode of treatment to patients with LSS and for those who did not respond to alternative modes of treatment. In other words, based on this study, patients with LSS can enjoy an enhanced quality of life with less pain and disability.

## References

[1] Deer T, Sayed D, Michels J (2019). A review of lumbar spinal stenosis with intermittent neurogenic claudication: disease and diagnosis. Pain Med.

[2] Ma XL, Zhao XW, Ma XJ (2017). Effectiveness of surgery versus conservative treatment for lumbar spinal stenosis: A systematic review and meta-analysis of randomized controlled trials. Int J Surg.

[3] Lafian AM, Torralba KD (2018). Lumbar spinal stenosis in older adults. Rheum Dis Clin North Am.

[4] Miscusi M, Trungu S, Forcato S (2018). Long-term clinical outcomes and quality of life in elderly patients treated with interspinous devices for lumbar spinal stenosis. J Neurol Surg A Cent Eur Neurosurg.

[5] Ishimoto Y, Kawakami M, Curtis E (2019). The Impact of lumbar spinal stenosis, knee osteoarthritis, and loss of lumbar lordosis on the quality of life: findings from the katsuragi low back pain study. Spine Surg Relat Res.

[6] Özdemir E, Paker N, Bugdayci D, Tekdos DD (2015). Quality of life and related factors in degenerative lumbar spinal stenosis: A controlled study. J Back Musculoskelet Rehabil.

[7] Haraldstad K, Wahl A, Andenæs R (2019). A systematic review of the quality of life research in medicine and health sciences. Qual Life Res.

[8] Motififard M, Naseri M, Panahi F, Teimouri M (2010). Is life easier and more pleasurable after total hip arthroplasty?. Shiraz E-Med J.

[9] Kobayashi Y, Ogura Y, Kitagawa T (2020). Gender differences in pre- and postoperative health-related quality of life measures in patients who have had decompression surgery for lumbar spinal stenosis. Asian Spine J.

[10] Jansson KA, Németh G, Granath F, Jönsson B, Blomqvist P (2009). Health-related quality of life (EQ-5D) before and one year after surgery for lumbar spinal stenosis. J Bone Joint Surg Br.

[11] Yüce İ, Kahyaoğlu O, Çavuşoğlu HA, Çavuşoğlu H, Aydın Y (2019). Long-term clinical outcome and reoperation rate for microsurgical bilateral decompression via unilateral approach of lumbar spinal stenosis. World Neurosurg.

[12] Eshaghi SR, Ramezani MA, Shahsanaee A, Pooya A (2006). Validity and reliability of the Short Form-36 Items questionnaire as a measure of quality of life in elderly Iranian population. Am J Appl Sci.

[13] Khor S, Lavallee D, Cizik AM (2018). Development and validation of a prediction model for pain and functional outcomes after lumbar spine surgery. JAMA Surg.

[14] Eneqvist T, Bülow E, Nemes S (2021). Does the order of total hip replacement and lumbar spinal stenosis surgery influence patient-reported outcomes: An observational register study. J Orthop Res.

[15] Hebert JJ, Abraham E, Wedderkopp N (2019). Patients undergoing surgery for lumbar spinal stenosis experience unique courses of pain and disability: A group-based trajectory analysis. PloS One.

[16] Zarghooni K, Beyer F, Papadaki J (2017). Quality of life and functional outcome after microsurgical decompression in lumbar spinal stenosis: a register study. Z Orthop Unfall.

[17] Pranata R, Lim MA, Vania R, July J (2020). Biportal endoscopic spinal surgery versus microscopic decompression for lumbar spinal stenosis: a systematic review and meta-analysis. World Neurosurg.

[18] Ma H, Hai B, Yan M, Liu X, Zhu B (2021). Evaluation of effectiveness of treatment strategies for degenerative lumbar spinal stenosis: a systematic review and network meta-analysis of clinical studies. World Neurosurg.

